# Unraveling the neuro-immune axis in cough hypersensitivity: from pathogenesis to synergistic management

**DOI:** 10.3389/fmed.2026.1824561

**Published:** 2026-08-04

**Authors:** Dingyi Shi, Jia He, Yajing Huang, Lisha Liu, Yunna Li, Qi Liu, Shunlin Peng

**Affiliations:** 1 Eye Health with Traditional Chinese Medicine Key Laboratory of Sichuan Province, School of Clinical Medicine, Chengdu University of Traditional Chinese Medicine, Chengdu, China; 2 Hospital of Chengdu University of Traditional Chinese Medicine, Chengdu, China; 3 First Teaching Hospital of Tianjin University of Traditional Chinese Medicine, Tianjin, China

**Keywords:** chronic cough, Cough Hypersensitivity Syndrome, immunization, neurogenic inflammation, refractory cough

## Abstract

Cough Hypersensitivity Syndrome (CHS) stands as the definitive pathological phenotype of refractory chronic cough, characterized by a sensory nervous system that over-reacts to even trivial, low-threshold stimuli. This condition is not merely a clinical symptom but a profound burden that erodes a patient’s quality of life. This narrative review synthesizes the latest breakthroughs in CHS pathogenesis and clinical management. We first unpack the neuro-immune dialogue within the airway microenvironment, detailing how a compromised epithelial barrier recruits alarmins to drive peripheral vagal sensitization via P2X3 and TRP ion channels. Moving beyond the periphery, we map the central sensitization cascade, where the remodeling of cortical inhibitory networks leads to a failure of the “cough brake.” To address the inherent clinical heterogeneity of CHS, we evaluate a necessary paradigm shift toward precision assessment–leveraging AI-powered acoustic monitoring and multidimensional, patient-reported outcomes to quantify the cough burden. Furthermore, we critically review the evolution of mechanism-based therapies, balancing the therapeutic promise of highly selective P2X3 antagonists against their safety profiles. Finally, we advocate for a multidisciplinary framework that marries targeted pharmacology with behavioral interventions like speech-language pathology (SLP), offering a forward-looking strategy for personalized care. This review aims to provide a systematic overview of the pathogenesis of CHS, with a particular focus on the neuro-immune axis, to offer a theoretical framework for personalized treatment, and to emphasize the importance of comprehensive, multidisciplinary diagnosis and treatment.

## Graphical Abstract

**Figure:**
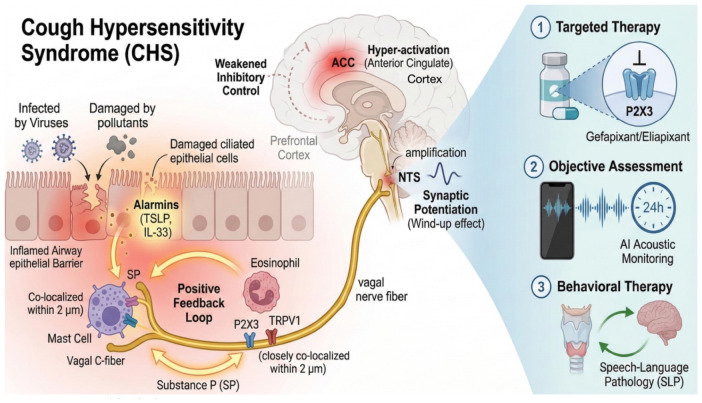
This schematic outlines the neuro-immune pathogenesis and precision management of Cough Hypersensitivity Syndrome (CHS). Epithelial injury releases alarmins (IL-33, TSLP) and ATP, activating peripheral vagal afferents via TRPV1/TRPA1/P2X3 channels, with further amplification by immune cells (mast cells, eosinophils). Persistent peripheral input drives central sensitization in the NTS and cortical regions, along with impaired descending inhibition (“cough brake” failure). Clinical assessment integrates AI-driven acoustic monitoring and patient-reported outcomes (LCQ). Targeted therapies are stratified by site of action—peripheral, dual, and central—alongside behavioral interventions (speech-language pathology). This integrated framework underscores a shift from empirical antitussives to mechanism-based, personalized care for CHS.

## 1 Introduction

Cough is a vital protective reflex for clearing airway secretions (1). When this reflex arc becomes abnormal, it can evolve into a severe chronic condition. Chronic cough, clinically defined as lasting more than 8 weeks in adults, has become a major global health challenge (2). Patients who have chronic cough suffer great pain in their body, psychology, and life. Most patients’ chronic cough is caused by some known reasons such as asthma, gastroesophageal reflux disease (GERD), or upper airway cough syndrome (UACS) (3, 4). However, a significant number of patients continue to cough severely despite optimal treatment for these underlying causes. We refer to this clinical problem as refractory chronic cough (RCC) or unexplained chronic cough (UCC) (5), which shows the inadequacy of conventional anatomical and pathophysiological concepts.

This gap in understanding about underlying mechanisms led to a paradigm shift in cough research. Researchers now recognize that the core nature of chronic cough lies in the hypersensitive state of airway sensory nerves (4). This realization led to the concept of Cough Hypersensitivity Syndrome (CHS). CC, RCC, and UCC are clinically distinct but share CHS as their underlying pathophysiological basis. RCC/UCC can be viewed as clinical presentations of CHS rather than merely progressive stages (6), with specific distinctions outlined in Table 1. Clinically, it features an exaggerated response to low-level inhaled triggers. Patients frequently report a persistent tickle or obstruction in the throat, along with secondary symptoms like chest tightness and dysphonia (7). Of particular importance is that this model focuses our attention on the role of internal neural sensitization; for example, inflammatory mediators produced locally in the airway (as seen during eosinophilic inflammation) lead to up-regulation of transient receptor potential channels (TRPs) such as transient receptor potential vanilloid 1 (TRPV1) and transient receptor potential ankyrin 1 (TRPA1) (8), which lower the activation threshold of peripheral sensory nerves. Additionally, there is a reduction in descending inhibition of the cough reflex and amplification of midbrain signaling (9). These findings confirm that CHS appears to be a systemic disease due to synergistic remodeling of peripheral and central neural circuits.

**TABLE 1:** Distinguishing clinical characteristics: CC vs. RCC/UCC vs. CHS.

Clinical concept	Core definition and pathological nature	Key clinical symptoms and manifestations	Common triggers
Chronic cough (CC)	Clinically defined as a cough lasting more than 8 weeks in adults (5).	Patients suffer great pain in their body, psychology, and life (5).	Usually caused by known underlying conditions such as asthma, gastroesophageal reflux disease (GERD), or upper airway cough syndrome (UACS) (3, 4).
Refractory / unexplained chronic cough (RCC/UCC)	A clinical problem where patients continue to cough severely despite optimal treatment for underlying causes (refractory), or they remain completely undiagnosed (unexplained) (5). Shows the inadequacy of conventional anatomical and pathophysiological concepts.	Severe, persistent coughing that does not resolve with standard therapies (5).	The triggers are either completely unknown, or the cough persists even when potential underlying triggers are optimally treated (5).
Cough Hypersensitivity Syndrome (CHS)	The definitive pathological phenotype of refractory chronic cough. It is an independent clinical entity driven by neurophysiological dysfunction, unifiying RCC and UCC (6). Its core nature lies in the hypersensitive state of airway sensory nerves.	Patients frequently report a persistent tickle or obstruction in the throat, along with secondary symptoms like chest tightness and dysphonia (7). It features an “intractable tickle,” an intense urge to cough, and a loss of the ability to voluntarily suppress the cough (failure of the “cough brake”)	Exaggerated response to trivial, low-threshold stimuli. Harmless triggers like cold air, talking, or deep breathing can provoke severe coughing (7).

In this review, we aim to systematically summarize the present status of CHS research. We first discuss the epidemiology and disease burden of CHS. Next, we analyze the role of “neuro-immune crosstalk” in mediating both peripheral and central sensitization. We also assess the available evidence regarding existing and emerging objective diagnostic tests. Furthermore, we review mechanism-based therapeutic interventions, comparing conventional neuromodulatory strategies, target-directed P2*3 therapeutics, and speech pathology therapy. By bridging bench research with multidisciplinary clinical management, this review provides a robust theoretical framework to advance the precise identification and personalized treatment of CHS.

## 2 Retrieval strategy

We searched PubMed and Web of Science for studies published between January 2010 and December 2025, using “Cough Hypersensitivity Syndrome (CHS)” as the core word and combined with words such as “epidemiology,” “pathogenesis,” “diagnosis,” and “treatment.” First, we screened the titles and abstracts of the articles, then read the full texts of those who met the requirements. At the same time, we manually searched the references of the included articles to find some classical high-quality studies.

Inclusion and exclusion criteria: We included peer-reviewed original articles, meta-analyses, and clinical trials published in English between January 2010 and December 2025, focusing on adult humans (aged ≥ 18 years). Exclusion criteria were case reports, editorials, conference abstracts, animal-only studies, and non-English publications. Study quality was assessed using the Cochrane Risk of Bias tool for randomized controlled trials and the AMSTAR checklist for meta-analyses. Because of the heterogeneity of outcomes and the lack of uniform diagnostic criteria across studies, a formal meta-analysis was not performed; instead, we conducted a narrative synthesis.

Four aspects of CHS were reviewed in this paper: epidemiology, pathogenesis, diagnosis, and treatment. Regarding the conflicting evidence, especially concerning the efficacy and safety of drugs, we adopted the results of the meta-analysis and high-quality clinical trials (such as COUGH-1 and COUGH-2). We emphasize that CHS should be diagnosed according to different neurological types, and at the same time consider factors such as the patient’s population, disease stage, and multi-disciplinary management model.

## 3 Epidemiology and burden

### 3.1 Epidemiology of CC

In 2015, the meta-analysis published by Song (10) is the most widely cited global epidemiological study on CC to date. Through a systematic search strategy, the study initially identified 17,891 citations; after screening, 398 full-text articles were retrieved, and 90 studies were finally included. The pooled analysis revealed a global total prevalence of CC of 9.6% (95% CI 7.6%–11.7%; I^2^ = 99%; 576,839 participants).

However, the prevalence of CC varies significantly across regions, ethnic groups, and genders, generally ranging from 2% to 18% (11). A prospective, randomized stratified study showed that the prevalence of CC among Canadian adults is approximately 16%–18% (11). In Chinese adults, the prevalence is approximately 6.0%–7.6%, with smoking, air pollution, and occupational exposure being common contributing factors (12). Data from the North American healthcare system estimate the community prevalence of CC among adults at 5%–10% (13). Several European countries report a prevalence of approximately 9%–11%, consistent with the global average (14).

Notably, multiple studies have identified female sex as a significant risk factor for CC. A retrospective study based on 11 cough clinics showed that among 10,032 registered patients with chronic cough, two-thirds (6,591) were female (mean age 55 years) (15). Similarly, a questionnaire survey of CC patients in the United Kingdom (with 373 valid responses returned) reported a mean patient age of 65.3 years (SD 12.0, range 9–88 years), with 73% being female and only 2% being current smokers (16).

### 3.2 Epidemiology of RCC/UCC and CHS

In contrast to CC, epidemiological data on RCC/UCC and CHS are very limited. Before 2015, the concepts of RCC/UCC were essentially absent from the literature, and consequently no related epidemiological data existed. Between 2015 and 2020, some publications began to mention RCC/UCC, but the data remained extremely scarce. Only the study by Haque et al. (17) reported that 42% of patients referred to a chronic cough clinic in the United Kingdom (*n* = 100) were diagnosed with RCC. Specific data on CHS in community settings are also lacking.

A recent study (18) using an observational design with national pharmacy and secondary care registries identified the proportion of possible chronic cough (PCC) among the Swedish adult population as 0.8%. When primary care registry data from a single healthcare region were added to the national cohort, the prevalence of PCC in that region increased to 1.8%, and a total of 44% of PCC patients met the diagnostic criteria for RCC. Overall, regional and even global epidemiological data on RCC, UCC, and CHS remain scarce.

### 3.3 Disease burden

The burden imposed by CHS and CC extends far beyond the symptoms themselves, profoundly affecting patients’ multidimensional health and societal resources. The impact on quality of life (QoL) is particularly severe and multifaceted: validated assessment tools such as the Leicester Cough Questionnaire (LCQ) demonstrate significant functional impairment across physical, psychological, and social domains, with effects comparable to those of severe chronic diseases such as chronic obstructive pulmonary disease (19). Studies have highlighted that CC significantly increases psychological distress (anxiety, depression) (20); CHS similarly leads to psychological depression, social impairment, and restricted physical activity (21). Because the brain regions processing cough signals overlap with emotion and stress centers, chronic CHS frequently induces anxiety, depression, and social withdrawal. These psychological factors can further lower the cough inhibition threshold, creating a vicious cycle of unpredictable and uncontrollable coughing (22, 23).

How to objectively capture and break this vicious cycle remains a key clinical challenge. In section “5 Advancements in clinical assessment,” we discuss emerging assessment tools–including AI-driven acoustic monitoring, objective cough monitors, and multidimensional patient-reported outcomes–that can help quantify both the peripheral and central components of this loop and guide mechanism-based interventions to restore descending inhibition.

The socioeconomic burden is equally substantial. Chronic cough ranks among the leading reasons for outpatient visits, with repeated consultations, diagnostic testing, and often ineffective treatments driving significant direct healthcare costs (24). Indirect costs arise from reduced labor productivity, absenteeism, early retirement, and social isolation experienced by many patients (14). The combined effects of high prevalence, significant personal suffering, and substantial economic costs indicate that chronic cough (and its refractory subtypes/Cough Hypersensitivity Syndrome) has become a major public health challenge requiring urgent attention. There is an urgent need to enhance clinical awareness and conduct targeted research to develop effective management strategies.

## 4 The pathophysiological core: airway microenvironment and neuro-Iimmune crosstalk

Cough hyper-responsiveness used to be considered a simple neural reflex, however, recent research has shown it is caused mainly by neuroimmune axis dysfunction in the airway microenvironment (25). Therefore, cough hyperreactivity is actually due to abnormal interaction between epithelial cells, infiltrating immune cells, and peripheral sensory nerves (26).

### 4.1 Impaired epithelial barrier and the release of alarmins

It is useful to distinguish two temporal phases in CHS pathogenesis: an initiation phase, often triggered by acute epithelial injury (e.g., viral infection), and a perpetuation phase, driven by chronic neuro-immune crosstalk involving eosinophils, mast cells, and persistent alarmin release.

**FIGURE 1:**
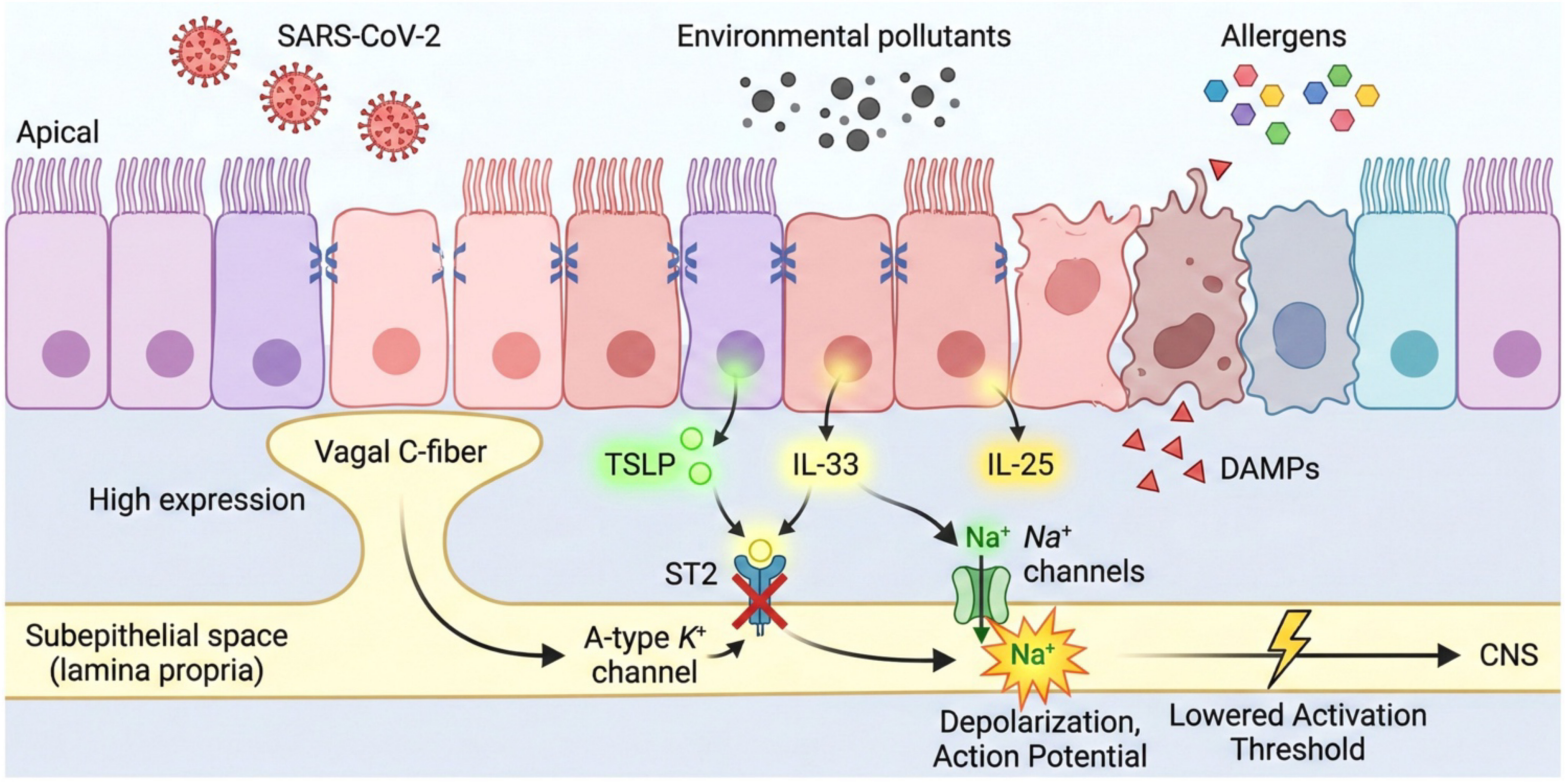
Airway epithelial barrier dysfunction and alarmin-mediated sensitization.

The airway epithelium is more than just a physical shield; it acts as a central biosensor that regulates both immune and neural responses. Song et al. (27) highlight viral infections, particularly SARS-CoV-2, as major triggers for chronic cough hypersensitivity. Even after the virus clears, the damaged epithelium continues to release damage-associated molecular patterns (DAMPs), driving prolonged “post-viral neuroinflammation.” Furthermore, when allergens or pollutants disrupt the epithelial barrier, these cells quickly release cytokines known as alarmins. Hansi et al. (28) showed that alarmins like thymic stromal lymphopoietin (TSLP), IL-33, and IL-25 act as upstream regulators. By binding to receptors such as TSLPR and ST2, they trigger inflammatory cascades and cause the abnormal overgrowth of airway sensory nerves. These alarmins serve a dual purpose. Besides recruiting immune cells, they directly target peripheral vagal sensory C-fibers, which heavily express their receptors. Specifically, Wang et al. (29) found that the IL-33/ST2 axis uses the Syk-p38β pathway to inhibit A-type potassium currents in dorsal root ganglion (DRG) neurons. Electrophysiological tests confirm that this directly boosts neuronal excitability. Ultimately, this means that even minor damage to the epithelial barrier translates directly into electrical signals, lowering the activation threshold of airway sensory nerves to mechanical and chemical stimuli. The mechanism diagram is shown in Figure 1.

### 4.2 Bidirectional crosstalk between immune cells and sensory networks

In CHS histopathology, immune cells (eosinophils, mast cells, and macrophages) closely colocalize with sensory nerve endings beneath the airway mucosa (30). This anatomical proximity provides the structural basis for bidirectional molecular signaling.

Tight anatomical coupling drives efficient neuro-immune interactions. Eosinophils promote nerve fiber growth during sensory remodeling (31), Drake et al. (32) demonstrated that eosinophils heavily aggregate around nerve fibers and parasympathetic ganglia in antigen-exposed airways. Similarly, mast cells act as crucial hubs linking the immune and nervous systems (33). While IgE-mediated mast cell activation boosts adaptive immunity, Bienenstock et al. (34) showed functional nerve-mast cell interactions involving substance P (SP), highlighting this axis as a key regulator of the airway microenvironment. Once activated, mast cells and eosinophils release high levels of type 2 inflammatory mediators (IL-4, IL-13, histamine, and leukotrienes). These mediators act as endogenous ligands that bind and phosphorylate neuronal ion channels (TRPV1, TRPA1, and P2*3), causing abnormal cation influx and high-frequency action potential firing (27). These mediators not only upregulate ion channel expression but also activate intracellular kinase cascades (e.g., MAPK, ERK, JAK-STAT) in vagal sensory neurons, leading to post-translational modification and membrane trafficking of channels, thereby lowering their activation threshold.

Macrophages also heavily influence nerve terminal polarization in CHS. Airway M2 macrophages drive nerve terminal sprouting by secreting brain-derived neurotrophic factor (BDNF) (35). BDNF and nerve growth factor (NGF), among other neurotrophic factors, mediate neuronal plasticity changes during airway inflammation (36, 37). *In vitro* and *in vivo* experiments by Braun et al. (36) confirmed that BDNF mediates neuronal hyperreactivity in mice following allergen exposure. Macrophages can also release exosomes carrying specific microRNAs, using extracellular vesicles (EVs) as core delivery vehicles to target sensory neurons (38). This non-canonical communication pathway offers a novel mechanistic explanation for CHS pathogenesis.

**FIGURE 2:**
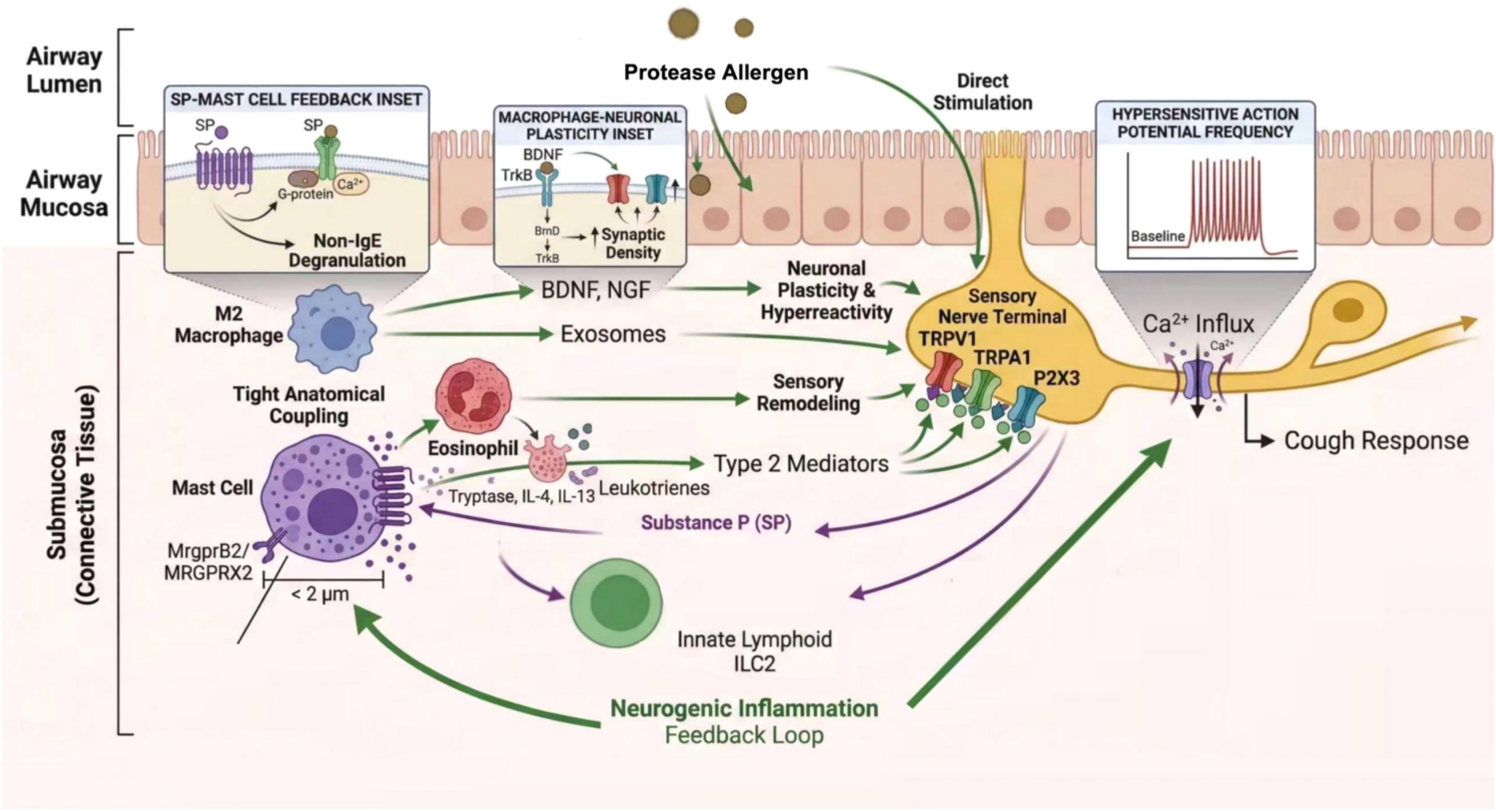
Immune-neural interactions in CHS pathogenesis.

Conversely, activated sensory C-fibers retrogradely release neuropeptides like SP and calcitonin gene-related peptide (CGRP). This triggers local vasodilation and immune cell degranulation (39, 40), creating a positive feedback loop known as “neurogenic inflammation.” This cascade sustains airway hypersensitivity and can lead to irreversible pathological changes. Tamari et al. (41) found that protease allergens (e.g., house dust mites) directly stimulate sensory neurons to release these neuropeptides. Nerve-derived SP can bind MrgprB2/MRGPRX2 receptors uniquely expressed on mast cells, triggering degranulation without relying on the classical IgE pathway (42). This neuro-mast cell synergy is anatomically evident in chronic cough biopsies, where sensory nerves and mast cells frequently lie less than 2 μm apart (34, 43). Furthermore, CGRP targets type 2 innate lymphoid cells (ILC2s) to amplify IL-5 and IL-13 release, which in turn recruits and activates airway eosinophils (6). Ultimately, this neuron-driven immune polarization sustains a highly pro-inflammatory local environment, further lowering the activation threshold of cough receptors. The mechanism of interaction between immune factors and nerves is illustrated in Figure 2.

### 4.3 Peripheral to central sensitization cascades

**FIGURE 3:**
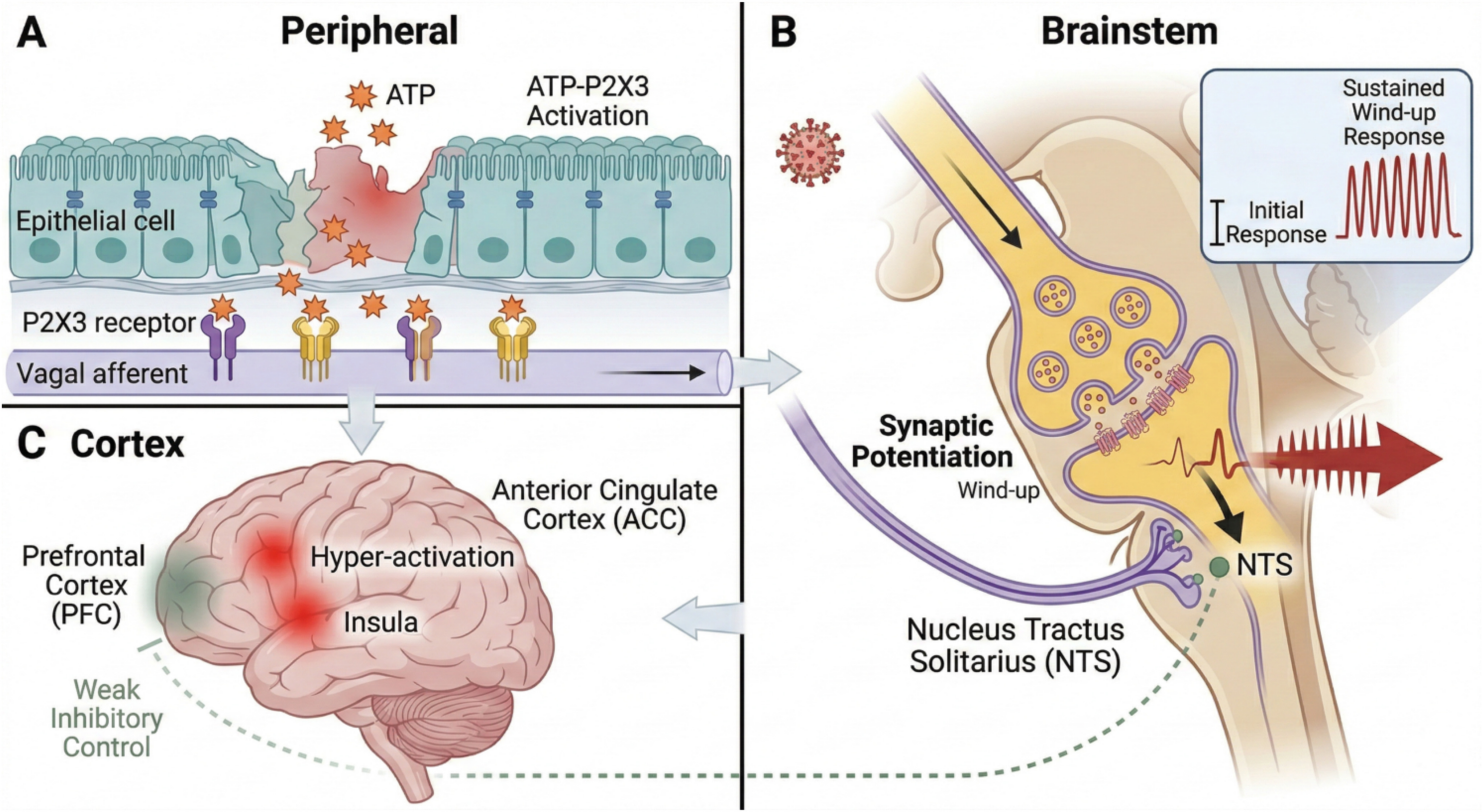
Peripheral to central sensitization cascades. **(A)** ATP activates P2X3 receptors. **(B)** Continuous erratic firing from peripheral nerves boosts synaptic transmission in excitatory NTS neurons, similar to the “wind-up” effect in chronic pain. **(C)** CHS patients have abnormally high activation in the anterior cingulate cortex (ACC), insula, and somatosensory cortex.

Peripheral sensitization drives the onset of Cough Hypersensitivity Syndrome (CHS), causing airway sensory neurons to fire spontaneously or overreact to mild stimuli. Molecularly, this stems from the upregulation and functional remodeling of key ion channels. However, CHS pathology extends beyond the periphery. It also involves amplified central nervous system (CNS) processing of sensory inputs and weakened descending inhibition. This central neuroplasticity ultimately causes the intense urge to cough and persistent symptoms. The peripheral-to-central cascade sensitization mechanism is illustrated in Figure 3.

#### 4.3.1 Peripheral sensitization and ion channel remodeling

Adenosine triphosphate (ATP) is a crucial extracellular signal released during airway epithelial damage or mechanical stress (44). ATP activates P2*3 receptors on vagal sensory nerves, triggering rapid depolarization and initiating the cough reflex. The clinical success of P2*3 antagonists, like Gefapixant, in reducing refractory cough frequency confirms this receptor’s central role in CHS (45). TRPV1 and TRPA1 are the primary multimodal nociceptors in the airways. TRPV1 responds to heat, acidity (pH < 6.0), and neuropeptide-driven phosphorylation (46), while TRPA1 detects mechanical pressure and chemical irritants like smoke (47). During CHS inflammation, neuro-immune mediators (e.g., IL-33, TSLP) heavily upregulate these channels on nerve terminals. This “molecular architectural remodeling” of ion channels elucidates the core mechanism by which harmless, non-specific stimuli–such as cold air, everyday speech, or mechanical traction from deep breathing–can trigger intense coughing episodes.

#### 4.3.2 Central sensitization and cortical network remodeling

Central sensitization in CHS occurs when the CNS overreacts to peripheral signals. Cough signals are amplified at brainstem synapses, particularly in the nucleus tractus solitarius (NTS) (48). As the primary relay station for the cough reflex, the medullary NTS undergoes significant synaptic plasticity. Central sensitization in the NTS is not solely neuronal; activated microglia and astrocytes release IL-1β, TNF-α, and BDNF, which enhance excitatory synaptic transmission and reduce inhibitory tone, amplifying cough signals before they reach cortical regions.

Continuous erratic firing from peripheral nerves boosts synaptic transmission in excitatory NTS neurons, similar to the “wind-up” effect in chronic pain (49–52). This chronic stimulation strengthens NTS synapses, meaning even weak peripheral signals are abnormally amplified before reaching the cortex. Clinically, this presents as exaggerated coughing in response to non-specific triggers (53–55).

Functional MRI (fMRI) reveals that CHS also involves extensive cortical network remodeling, rather than just brainstem dysfunction. Ando et al. (51) used fMRI to show that upon cough stimulation, CHS patients have abnormally high activation in the anterior cingulate cortex (ACC), insula, and somatosensory cortex. This cortical hypersensitivity aligns with the “intractable tickle” and intense urge to cough that patients report. Normally, the prefrontal cortex actively inhibits the brainstem cough reflex. In CHS, however, this descending suppression network weakens. Without this regulatory circuit, patients lose the ability to voluntarily suppress their cough. The failure of this “central braking system,” combined with peripheral sensitization, drives the full pathology of CHS (49, 56).

The disruption of this central braking system will be addressed in section “5 Advancements in clinical assessment,” which reviews objective and subjective assessment tools, and in section “6 Mechanism-based targeted therapies: efficacy and controversies,” which details mechanism-based therapies aimed at restoring descending inhibition.

## 5 Advancements in clinical assessment

### 5.1 Limitations of traditional diagnostic strategies

Current clinical diagnosis relies primarily on medical history and adjunctive challenge tests, though their limitations are increasingly apparent. While inhalation challenges (e.g., capsaicin or ATP) assess the cough reflex, they lack standardized protocols. Furthermore, they cannot replicate complex, real-world physical stimuli like cold air or mechanical stretch, severely limiting their clinical utility (57, 58). Although the capsaicin inhalation test is safe and reproducible for studying chronic cough, its usefulness for management and differential diagnosis is limited (59). Similarly, ATP challenge has been shown to produce tussive responses mediated through P2*3 purinergic receptors, but this acute effect does not sufficiently explain Cough Hypersensitivity Syndrome (60). Subjective tools also present challenges. The visual analogue scale (VAS) is commonly used, but it is heavily influenced by patient mood and transient perceptions (61). This high inter-individual variability prevents its use as a sole primary endpoint in clinical trials. Similarly, while patient-reported outcome (PRO) questionnaires like the Leicester Cough Questionnaire (LCQ) are well-validated for assessing QoL, they rely on patient memory, introducing recall bias regarding actual cough frequency (19).

In addition to subjective questionnaires, dedicated objective cough monitors have been developed. The VitaloJAK™ system and the Leicester Cough Monitor (LCM) are non-invasive, ambulatory devices that record cough sounds via acoustic sensors applied to the suprasternal notch, providing objective 24-h cough frequency counts. A recent systematic review of 54 studies (4,909 patients) identified VitaloJAK as the most evaluated objective cough monitoring system (19 studies), followed by LCM (18 studies) (62). However, the labor-intensive analysis required for manual annotation and the limited correlation with patient-reported outcome measures remain barriers to routine clinical use.

### 5.2 AI acoustic monitoring and objective data integration

To overcome the limitations of subjective testing, AI-driven acoustic monitoring is emerging as a promising tool in clinical research and is increasingly used as an objective endpoint in trials. Using smartphone applications and wearable acoustic sensors, clinicians can continuously collect 24-h audio data in patients’ natural environments (63). Deep learning algorithms then automatically extract time- and frequency-domain features of cough sounds (e.g., fundamental frequency, energy distribution, and burst duration). This allows for the precise quantification of both cough frequency and intensity (63–65).

Multiple noteworthy studies have advanced this field. A deep-learning-based cough detector using SqueezeNet in conjunction with wavelet scalograms achieved an accuracy of 92.21% and precision of 95.59% for distinguishing intentional coughing from speech, laughter, or throat noises (66). An end-to-end deep learning system, the “Neural Cough Counter,” takes raw audio signals as input and achieves cough event detection error-rate scores of 0.31 and 0.32 on both in-house and public datasets, with the lowest average hourly symmetric mean absolute error (8.48%) compared to the Leicester Cough Monitor and XGBoost (67). More recently, an ensemble deep fusion model combining CNN, MLP, and RNN architectures achieved up to 99.94% accuracy in classifying audio recordings into unhealthy coughs, healthy coughs, and non-cough sounds (68). These objective metrics accurately capture circadian patterns, particularly highlighting the impact of nocturnal coughing on sleep quality. However, due to high device costs, lack of algorithm standardization, and time burden, it has not yet become a routine standard in clinical practice (64).

### 5.3 The value of multidimensional patient-reported outcomes

Despite advances in objective technologies, subjective patient ratings remain indispensable. A multidimensional PRO assessment must capture not only cough frequency but also physical, psychological, and social impairments (24).

Importantly, cross-validation of 24-h objective cough data from monitors like VitaloJAK or AI-based tools with subjective LCQ and VAS scores can help build a comprehensive CHS phenotyping model (69). Such integration allows clinicians to identify distinct clinical phenotypes, such as “high cough frequency, low psychological burden” versus “low cough frequency, high quality-of-life impairment” (4). Ultimately, this multidimensional approach facilitates highly targeted, personalized intervention strategies (57, 69).

### 5.4 Assessing the neuro-immune axis in clinical settings

Although growing evidence suggests that neuro-immune interactions are a driving force behind CHS, their direct clinical assessment remains in the early stages of exploration. Several potential methods may help fill this gap. Measuring soluble mediators such as substance P (SP), calcitonin gene-related peptide (CGRP), brain-derived neurotrophic factor (BDNF), and nerve growth factor (NGF) in induced sputum, bronchoalveolar lavage fluid, or plasma may serve as markers of neuroimmune activation. Immunohistochemical analysis indicates that, in animal models, elevated expression of SP and CGRP in lung tissue correlates with cough severity (70), and similar dysregulation of neuropeptides has also been observed in patients with post-infectious cough. Concurrently, functional imaging has revealed central sensitization in chronic cough syndrome (CHS). Using functional magnetic resonance imaging (fMRI), Ando et al. demonstrated that cough sensitization occurs concurrently with increased neural activity in the midbrain (including the cuneus and periaqueductal gray), whereas cough suppression is associated with reduced activity in forebrain networks involving the dorsomedial prefrontal cortex and anterior cingulate cortex (9). Similarly, cough-evoked potentials or quantitative sensory testing can provide electrophysiological indicators of central sensitization. Although bronchial biopsy is invasive, the histological colocalization of mast cells and eosinophils with nerve fibers provides direct evidence of neuro-immune structural interactions (34, 41). Future research should prioritize the development of standardized neuroimmune biomarker panels to enable routine clinical phenotyping.

## 6 Mechanism-based targeted therapies: efficacy and controversies

**FIGURE 4:**
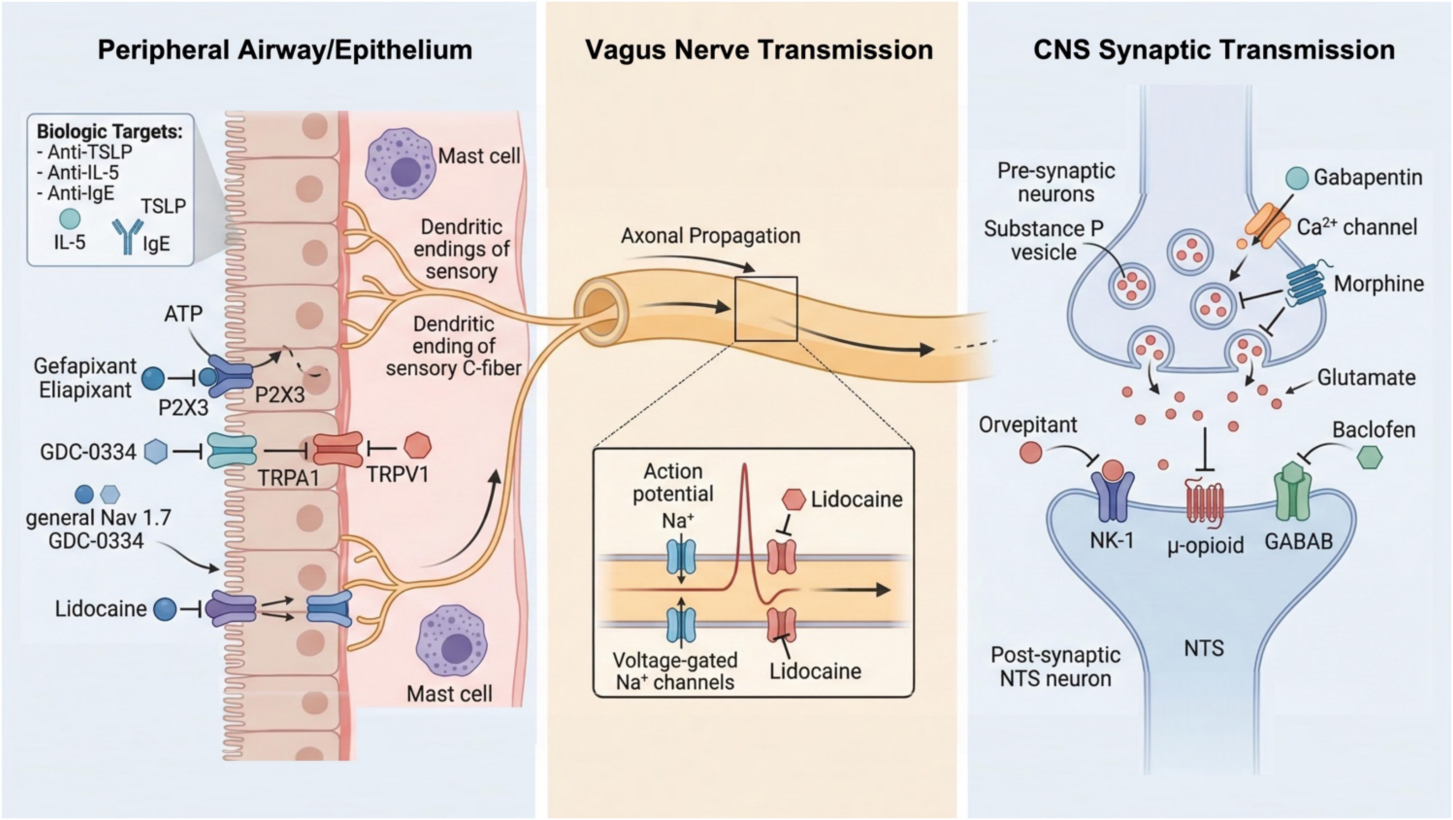
Pharmacological mechanisms of neuromodulators and non-specific targeted drugs.

Deep understanding of CHS pathophysiology has enabled precise targeted interventions. Current treatment strategies are shifting from non-specific antitussives to individualized, neuroscience-based therapies. The specific pharmacological mechanism diagram is shown in Figure 4. The advantages and disadvantages of various targeted therapies are summarized in Table 2.

**TABLE 2:** The advantages and disadvantages of various targeted therapies.

Drug class / representative drugs	Mechanism / target	Pros	Cons
P2X3 receptor antagonists (Gefapixant, Eliapixant)	Targeting peripheral mechanisms; blocks ATP-P2X3 receptor activation.	Clinically validated to significantly reduce cough frequency in chronic cough patients; highly selective antagonists (e.g., Eliapixant) have shown improved tolerability.	Off-target inhibition leads to dysgeusia (taste-related adverse events), which is highly dose-dependent (up to 58%–69% for high-dose Gefapixant); variable patient responses.
TRPV1 antagonists (e.g., SB-705498)	Targeting peripheral mechanisms; blocks heat, acid, and neuropeptide-driven sensitization.	Inhaled formulations significantly improve cough reflex sensitivity to capsaicin within 2 h.	Early systemic TRPV1 antagonists caused hyperthermia side effects; inhaled SB-705498 demonstrated no significant effect on cough severity, urge-to-cough, or cough-specific quality of life.
TRPA1 antagonists (GDC-0334, GRC-17536)	Targeting peripheral mechanisms; blocks mechanical pressure and chemical irritant detection.	Showed potential in reducing allergen-induced airway inflammation, edema, and chemically-induced cough in preclinical animal models; highly potent with excellent oral bioavailability.	Current efficacy evidence is primarily derived from early *in vivo* studies and animal models.
Inhaled lidocaine	Targeting peripheral mechanisms; non-specific voltage-gated sodium channel (Nav) blocker.	Effectively suppresses chemically-induced cough (citric acid and capsaicin); provides rapid cough suppression (within 15 min) for refractory cough in end-of-life care.	Mild, transient side effects include oropharyngeal numbness and bitter taste; can cause bronchoconstriction in some asthma patients.
Central neuromodulators (gabapentin, pregabalin)	Targeting central mechanisms; suppresses central nervous system hypersensitivity.	Irreplaceable for CHS patients with prominent central sensitization features; significantly improves cough-specific quality of life.	Effectiveness is often limited; readily causes central nervous system side effects such as drowsiness, dizziness, nausea, and fatigue; carries high risks of addiction, abuse, and misuse.
Mu-opioid receptor agonists (low-dose morphine)	Targeting central mechanisms; directly inhibits excitatory synaptic transmission in the NTS.	Significantly reduces cough frequency by approximately 30%–40% in refractory patients; alleviates anxiety related to unpredictable cough episodes.	Considered an “end-line intervention” strictly for severe CHS patients with profoundly impaired QoL who are unresponsive to conventional therapies.
NK-1 receptor antagonists (orvepitant)	Dual targeting (peripheral and central mechanisms); disrupts the positive feedback loop of neurogenic inflammation.	Peripherally exerts anti-inflammatory/desensitizing effects; centrally modulates synaptic transmission to increase the cough threshold; provides statistically and clinically significant improvements in objective daytime cough frequency with a good safety and tolerability profile.	Lack of large-scale clinical data to support.
GABA receptor agonists (baclofen)	Dual targeting (peripheral and central mechanisms).	Increases the cough reflex threshold; also reduces transient lower esophageal sphincter relaxations, providing synergistic clinical value for CHS patients with concomitant GERD.	Central side effects like dizziness and muscle weakness limit widespread clinical use.
Emerging biologics (Anti-IgE, Anti-IL-5, anti-TSLP/IL-4R)	Upstream blockade of neuro-sensitization at its source.	Offers precise, novel interventions to disrupt core neuro-immune interactions for specific CHS subsets.	Exploratory value only; limited to patients exhibiting marked T2 airway inflammation (e.g., high FeNO levels or elevated eosinophil counts).

### 6.1 Targeting peripheral mechanisms

#### 6.1.1 P2*3 receptor antagonists

The clinical validation of P2*3 receptor antagonists represents a major breakthrough in RCC and UCC treatment over the past decade. Gefapixant, the first globally approved drug in this class, demonstrated significant reduction in cough frequency in chronic cough patients during the COUGH-1 and COUGH-2 phase III trials, confirming its clinical efficacy (45). Despite its notable effectiveness, the most prominent side effect is dysgeusia. In these phase III trials, 11%–20% of patients receiving 15 mg gefapixant twice daily reported taste-related adverse events, primarily dysgeusia. This proportion increased to 58%–69% in the 45 mg twice-daily group (71). This side effect results from off-target inhibition of P2*2/3 heteromers, which mediate taste signal transmission (45, 72). Variable patient responses further underscore the heterogeneity of CHS. To improve tolerability, highly selective P2*3 antagonists such as Eliapixant and Sivopixant are under development (73, 74). Morice et al. (75) in a parallel randomized, placebo-controlled, double-blind study, confirmed that Eliapixant significantly reduced cough frequency and severity. In this study, 45 mg gefapixant administered twice daily inhibited both P2*3 and P2*2/3 receptors. At week 12, awake cough frequency was reduced by 18% compared to placebo; at week 24, the reduction was 16%. Twenty-four-hour cough frequency reductions versus placebo were 18% and 15%, respectively. Taste-related adverse events were reported by 8%–10% of participants, and this symptom was dose-independent. Dicpinigaitis et al. (76) conducted a 12-week randomized trial involving 310 participants receiving 25 mg, 75 mg, or 150 mg twice daily. Results showed that compared to the placebo group, more subjects in the 75 mg Eliapixant group achieved the following endpoints at week 12: ≥30% reduction in 24-h cough count from baseline and ≥30 mm reduction in cough severity score. However, no improvement in cough-related QoL was observed, as measured by LCQ total score. In the 150 mg Eliapixant group, 24% of subjects reported taste-related adverse events; those receiving lower doses reported fewer adverse events.

#### 6.1.2 TRPV1 and TRPA1 antagonists

Transient receptor potential channel family members are highly expressed in sensing heat, acid, and mechanical stimuli. They constitute an important basis for mediating peripheral sensitization in CHS. Due to TRPV1’s systemic role in thermoregulation, early systemic TRPV1 antagonists often caused hyperthermia as a side effect (77). This finding alerted researchers that airway nerve terminal sensitization often involves systemic homeostatic receptors. Current research focus has shifted to inhaled antagonists, aiming for localized airway targeting. Results from an randomized controlled trial (RCT) by Khalid et al. (78) showed that SB-705498 treatment significantly improved cough reflex sensitivity to capsaicin within 2 h. Marginal significant improvement compared to placebo was observed at 24 h. However, SB-705498 demonstrated no significant effect on cough severity, urge-to-cough, or cough-specific quality of life. For TRPA1 antagonists sensitive to chemical stimuli such as cigarette smoke, GRC-17536 (70) showed potential in reversing citric acid-induced cough in guinea pigs during early *in vivo* studies. GDC-0334 is a highly potent, selective small molecule inhibitor with excellent oral bioavailability. In a guinea pig model, Balestrini et al. (79) demonstrated that oral GDC-0334 dose-dependently reduced ammonia chloride-induced cough counts and prolonged the latency to first cough. These preclinical findings suggest a potential inhibitory effect of TRPA1 antagonism on allergen-induced airway inflammation and cough, although human data are lacking.

#### 6.1.3 Inhaled lidocaine

Voltage-gated sodium channels (Nav) are critical for action potential generation in neurons. Inhaled lidocaine acts as a non-specific sodium channel blocker. Research by Svajdova et al. (80) investigated its effect on citric acid and capsaicin-induced cough in awake naive guinea pigs. During citric acid (0.8 M) cough challenge, pre-inhalation and continuous nebulization of lidocaine (10 mM) effectively suppressed cough responses (cough count). Similarly, lidocaine reduced cough counts during capsaicin (50 μM) challenge. Nebulized lidocaine is also used for refractory cough in end-of-life care to improve patient comfort (81). Nebulized lidocaine (1%–4%) demonstrated rapid cough suppression (within 15 min) in 70% of cancer patients, with effects lasting 2–4 h. Mild side effects included oropharyngeal numbness (15%) and bitter taste (10%), all transient. However, 25% of asthma patients experienced bronchoconstriction (FEV1 decline ≥ 15%), which was reversed by bronchodilators. Novel, highly selective Nav1.7 or Nav1.8 inhibitors are currently under development (82). These drugs aim to directly reduce conduction efficacy in sensory axons, thereby blocking pathological neuronal discharge triggered by alarmins released upon epithelial damage.

### 6.2 Targeting central mechanisms

Managing refractory cough has shifted toward targeting the neural dysregulation driving cough hypersensitivity. The cough reflex operates via a neural arc initiating from peripheral vagal afferents, propagating through brainstem second-order sensory neurons, and integrating within the cerebral cortex. Interrupting this peripheral-central cascade across specific anatomical nodes underpins contemporary pharmacological strategies (83).

#### 6.2.1 Gabapentin and pregabalin

Centrally acting drugs remain irreplaceable for CHS patients with prominent central sensitization features. Gabapentin and pregabalin are commonly used clinically to suppress central nervous system hypersensitivity. Ryan et al. (84) conducted a randomized double-blind trial. 62 patients were assigned to gabapentin (*n* = 32) or placebo (*n* = 30). Results showed gabapentin significantly improved cough-specific quality of life. Ten patients (31%) in the gabapentin group experienced adverse events (most commonly nausea and fatigue), compared with 3 patients (10%) in the placebo group. Saint-Pierre conducted a prospective cohort study enrolling 50 patients (85). Participants received 75 mg of pregabalin orally at bedtime for 4 weeks, followed by a switch to 75 mg twice daily. Results showed that 56% of patients demonstrated a significant improvement in their LCQ total score. Patients who responded to pregabalin treatment were more likely to have refractory chronic cough (associated with underlying pulmonary disease) rather than chronic cough of unknown origin (*p* = 0.01). Patients with significant symptom improvement had more severe baseline symptoms. Although these medications demonstrate some efficacy in empirical treatment, their effectiveness is often limited. They are prone to causing central nervous system side effects such as somnolence and dizziness. More importantly, these medications carry a high risk of addiction, abuse, and misuse. Since most clinical data originate from pilot studies with small sample sizes or significant patient heterogeneity, these medications are generally recommended only for specific patient groups that do not respond to conventional therapies (86).

Mechanistically, they bind to the α2-δ subunit of voltage-gated calcium channels, reducing the release of excitatory neurotransmitters (e.g., glutamate, substance P) in the brainstem and thereby attenuating central amplification of cough signals (83). However, emerging evidence highlights heterogeneous responses and significant risks, particularly in vulnerable populations, with off-label use in children supported only by low-quality evidence (83). Given the high prevalence of anxiety and depression in CHS, the potential for addiction and misuse further restricts their role to second- or third-line specialist therapy (85). Future research should prioritize predictive biomarkers to enable precision prescribing for the most responsive patient subsets.

#### 6.2.2 Mu-opioid receptor agonists

For severe CHS patients with profoundly impaired QoL and unresponsiveness to conventional therapies, low-dose potent opioids are considered “end-line interventions.” Mu-opioid receptor agonists directly inhibit excitatory synaptic transmission evoked by peripheral afferents in the NTS (87). Multiple randomized controlled trials confirm that low-dose morphine (5–10 mg twice daily) significantly reduces cough frequency by approximately 30%–40% in refractory patients and alleviates anxiety related to unpredictable cough episodes (88).

### 6.3 Dual targeting: peripheral and central

#### 6.3.1 NK-1 receptor antagonists

NK-1 receptor antagonists disrupt the positive feedback loop of neurogenic inflammation. In the peripheral airway microenvironment, they exert anti-inflammatory and desensitizing effects. On the one hand, activated C fibers retrogradely release neuropeptides such as substance P. Substance P binds to NK-1 receptors on the surfaces of immune cells, like mast cells, inducing degranulation and inflammatory mediator release. This leads to local vasodilation and tissue edema. NK-1 antagonists block this pathway, preventing the long-term perpetuation and potential irreversibility of airway hypersensitivity. On the other hand, NK-1 antagonists increase the cough threshold by modulating synaptic transmission. Substance P is a key neurotransmitter for signal transmission in second-order neurons within the nucleus tractus solitarius (NTS). NK-1 antagonists interrupt this signaling cascade, effectively inhibiting synaptic gain amplification and restoring impaired descending inhibition, thereby reducing the urge to cough (89, 90). The NK-1 receptor antagonist Orvepitant (91) acts across both dimensions. It peripherally blocks neuropeptide-induced immune cell activation and centrally inhibits signal amplification in the brainstem. Thirteen patients with a daytime cough frequency between 3 and 250 coughs/hour received 30 mg of Orvepitant daily for 4 weeks. Twenty-four-hour objective cough frequency was measured at baseline and at weeks 1, 4, and 8. The primary endpoint was the change in daytime cough frequency from baseline at week 4. The baseline mean cough frequency was 71.4 coughs/hour. At week 4, a statistically and clinically significant improvement in objective daytime cough frequency was observed: an 18.9 coughs/hour (26%) reduction from baseline. This effect appeared by week 1 and persisted at week 8 after treatment cessation. Cough severity VAS scores and QoL also showed statistically significant improvements. Although the final statistical difference in objective cough frequency improvement was not significant in this study, the results indicated that Orvepitant had a good safety and tolerability profile. In an RCT targeting chronic cough patients with lung cancer, NK-1 receptor antagonists significantly reduced cough frequency and severity while improving cough-specific quality of life (92).

#### 6.3.2 GABA receptor agonists

Baclofen, a GABAB receptor agonist, alleviates hypersensitivity through dual central and peripheral mechanisms. Baclofen primarily acts on the medullary cough reflex center. It increases the cough reflex threshold by inhibiting the release of excitatory neurotransmitters, such as glutamate. Beyond its direct antitussive effects, baclofen reduces transient lower esophageal sphincter relaxations. This offers synergistic clinical value for CHS patients with concomitant GERD features (93). Xu et al. evaluated changes in cough symptom scores, capsaicin threshold, reflux symptom scores, and adverse effects in 16 patients after 8 weeks of treatment with 20 mg of baclofen three times daily. Cough symptoms resolved or improved in 56.3% (9/16) of the patients. This included 6 patients with acid reflux-induced cough (66.7%) and 3 with non-acid reflux-induced cough (33.3%). After baclofen treatment, cough symptom scores began decreasing at week 2, significantly decreased by week 6, and reached their nadir at week 8. At the end of treatment, the minimum capsaicin concentrations inducing ≥2 and ≥5 coughs increased significantly from 0.98 (1.46) to 1.95 (6.82) μmol/L and from 1.95 (7.31) to 7.8 (13.65) μmol/L, respectively. Reflux symptom scores decreased from 8.0±1.6 to 6.8±0.8. The acid reflux episode frequency was significantly lower in responders than in non-responders (94). Despite its efficacy in certain refractory cases, central side effects like dizziness and muscle weakness limit its widespread clinical use.

### 6.4 Upstream blockade: emerging biologics

For specific CHS phenotypes, biologics offer a novel approach to block neuro-sensitization at its source. For patients exhibiting marked T2 airway inflammation, such as high fractional exhaled nitric oxide (FeNO) levels or elevated eosinophil counts, targeted biologics demonstrate exploratory value (7). Current clinical explorations include anti-IgE antibodies, anti-IL-5 therapies, and biologics targeting TSLP or IL-4R. By disrupting core neuro-immune interactions involving epithelial-derived cytokines, these therapies may provide precise interventions for specific CHS subsets.

### 6.5 Multitarget potential of natural products in CHS modulation

Research by Laude et al. (95) confirmed that menthol-containing aromatic vapors inhibited the cough reflex in awake guinea pigs. Menthol exerts antitussive effects primarily by activating the cold receptor TRPM8. TRPM8 activation can elicit a “cross-inhibitory” effect, suppressing TRPV1- and TRPA1-mediated cough signal transmission at the central or peripheral levels. Experiments by Beltrán et al. (96) demonstrated that 6-gingerol dose-dependently inhibits TASK-1, TASK-3, and TRESK potassium channels, which are highly expressed in airway sensory neurons. By modulating the neuronal resting membrane potential, it directly suppresses the abnormal discharge and activation of airway sensory neurons. Lee et al. (97) found that fermented *Platycodon grandiflorus* extract, which features an increased platycodin D content due to fermentation, exhibited neuroprotective and anti-sensitization effects. It significantly reduced eosinophil and total cell counts in the bronchoalveolar lavage fluid of LPS/OVA-induced asthmatic mice, alleviating airway inflammation and cough reflex sensitivity *in vivo*.

## 7 Discussion

This review comprehensively and narratively synthesizes emerging evidence on the complex neuro-immune interactions in CHS. Although we employed a structured literature search and predefined inclusion/exclusion criteria (see section “3 Epidemiology and burden”), the heterogeneous methodologies and outcome measures across studies precluded a formal systematic review or meta-analysis. Current consensus recognizes CHS not merely as a simple physical airway response to external stimuli, but as a systemic neuropathophysiological process involving epithelial barrier damage, peripheral vagal nerve sensitization, and central cortical network remodeling. Across epidemiology, diagnosis, and treatment, recent data consistently demonstrate that CHS imposes a substantial socioeconomic burden and profoundly impairs patient quality of life. Its heterogeneous clinical manifestations demand a shift from empirical antitussive approaches toward mechanism-based targeted therapies guided by precise phenotypic stratification. This review differs from previous publications in several aspects: it emphasizes the spatiotemporal dynamics of neuro-immune crosstalk, integrates recent advances in AI-based monitoring, and systematically evaluates both peripheral and central mechanisms of sensitization within a unified framework.

### 7.1 Clinical perspective

**FIGURE 5:**
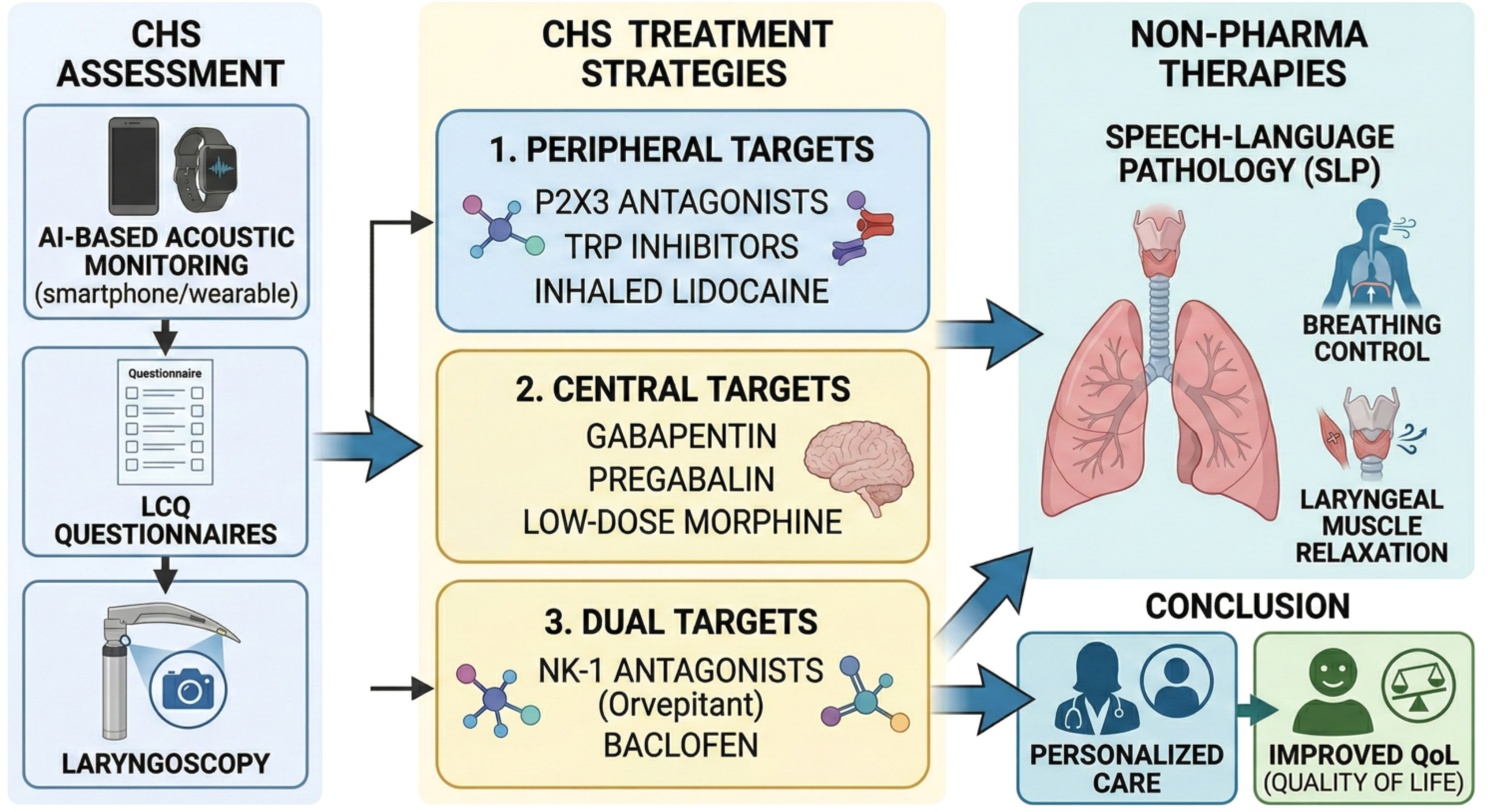
Multidimensional clinical management facilitates the realization of precision medicine at CHS.

From a clinical standpoint, these insights support the establishment of more proactive multidisciplinary team pathways. Respiratory specialists, speech therapists, and psychologists should collaboratively identify high-risk patients. Laryngoscopy plays a key role in evaluating patients with cough hypersensitivity, helping to identify comorbidities such as inducible laryngeal obstruction or vocal cord dysfunction (98). By recognizing anatomical and physiological abnormalities in laryngeal function, clinicians can develop precise behavioral interventions to alleviate the urge to cough from an upper airway protection perspective. Given the high overlap between CHS and comorbidities like GERD, anxiety, and depression, single-drug approaches often prove insufficient. SLP has been validated as an effective means to reverse central sensitization. Through physical interventions such as vocal exercises, breathing control, and laryngeal muscle relaxation, patients can re-establish voluntary control over the cough reflex. Clinical evidence indicates that such non-pharmacological interventions offer significant advantages in improving the patient’s subjective experience and reducing the objective cough frequency (99). The flowchart is shown in Figure 5.

While our review primarily focuses on adult CHS, it is crucial to acknowledge that cough hypersensitivity and impaired airway clearance present formidable challenges in pediatric populations and patients with neuromuscular disorders (NMDs). In these groups, the pathophysiology is often compounded by mechanical respiratory insufficiency, bulbar dysfunction, and impaired mucociliary clearance, rather than isolated neuro-immune sensitization. Consequently, the therapeutic paradigm shifts from neuromodulation toward a comprehensive strategy of lung function preservation and active cough augmentation. Standard-of-care interventions in pediatric NMDs include mechanical insufflation-exsufflation (MI-E) devices, manual assisted coughing, and tailored respiratory physiotherapy, all aimed at optimizing lung volumes and preventing atelectasis and pneumonia (100). These approaches have been shown to significantly reduce respiratory exacerbations and improve survival in conditions like spinal muscular atrophy and Duchenne muscular dystrophy. Recognizing this continuum–where the primary goal is to manage the mechanical consequences of a weak or ineffective cough–is essential for a holistic view of cough care. This perspective underscores the need for multidisciplinary teams to bridge the gap between neurophysiological hypersensitivity and biomechanical cough failure, ensuring that vulnerable patient subgroups benefit from integrated respiratory and rehabilitative care.

### 7.2 Research perspective

From a research perspective, several major knowledge gaps remain. First, despite the excellent efficacy of P2*3 antagonists, the off-target effect of dysgeusia remains a primary bottleneck limiting their widespread adoption. The development of highly selective subunit inhibitors or biomarkers predicting side effects is urgently needed. Second, the long-term evolution mechanisms of post-COVID cough and the persistent role of epithelial-derived DAMPs remain unclear. Future research should explore genetic associations between specific inflammatory phenotypes and neuropeptide levels to enable genomics-based individualized risk identification.

### 7.3 Limitations

The conclusions of this review must be interpreted in light of its limitations. Most epidemiological data exhibit significant geographic bias, primarily concentrated in North America, Europe, and China, potentially underrepresenting global population diversity. Regarding objective assessment, despite the promise of AI acoustic monitoring, the field still lacks standardized algorithms and reference values. Additionally, this is a narrative rather than a systematic review with a focused literature scope, potentially omitting key findings from non-English sources. Evaluations of different interventions reflect synthesized assessments based on available RCT evidence rather than a formal meta-analysis.

## 8 Conclusion and future directions

Cough Hypersensitivity Syndrome (CHS) is a systemic dysfunction driven by epithelial barrier damage, neuro-immune dysregulation, and central sensitization. Traditional anatomical models and conventional antitussives are insufficient, leaving patients with poor quality of life. This review advocates for precise phenotyping and objective assessment in future management. Key challenges remain: lack of predictive biomarkers for P2*3 antagonists, and unresolved long-term outcomes of post-COVID cough hypersensitivity. Integrating pharmacological with non-pharmacological interventions to reshape neural circuits requires a clinical shift from external triggers to internal neuropathological remodeling. Future research should prioritize biomarkers and large-scale trials to interrupt neurogenic inflammation and improve patients’ quality of life.
